# Inflation targeting: A time-frequency causal investigation

**DOI:** 10.1371/journal.pone.0295453

**Published:** 2023-12-11

**Authors:** Tanweer Ul Islam, Dajeeha Ahmed

**Affiliations:** Department of Economics, National University of Sciences and Technology, Islamabad, Pakistan; University of City Island, CYPRUS

## Abstract

The enduring discourse regarding the effectiveness of interest rate policy in mitigating inflation within developing economies is characterized by the interplay of structural and supply-side determinants. Moreover, extant academic literature fails to resolve the direction of causality between inflation and interest rates. Nevertheless, the prevalent adoption of interest rate-based monetary policies in numerous developing economies raises a fundamental inquiry: What motivates central banks in these nations to consistently espouse this strategy? To address this inquiry, our study leverages wavelet transformation to dissect interest rate and inflation data across a spectrum of frequency scales. This innovative methodology paves the way for a meticulous exploration of the intricate causal interplay between these pivotal macroeconomic variables for twenty-two developing economies using monthly data from 1992 to 2022. Traditional literature on causality tends to focus on short- and long-run timescales, yet our study posits that numerous uncharted time and frequency scales exist between these extremes. These intermediate scales may wield substantial influence over the causal relationship and its direction. Our research thus extends the boundaries of existing causality literature and presents fresh insights into the complexities of monetary policy in developing economies. Traditional wisdom suggests that central banks should raise interest rates to combat inflation. However, our study uncovers a contrasting reality in developing economies. It demonstrates a positive causal link between the policy rate and inflation, where an increase in the central bank’s interest rates leads to an upsurge in price levels. Paradoxically, in response to escalating prices, the central bank continues to heighten the policy rate, thereby perpetuating this cyclical pattern. Given this observed positive causal relationship in developing economies, central banks must explore structural and supply-side factors to break this cycle and regain control over inflation.

## 1. Introduction

There is a long-standing debate among economists about the effectiveness of interest rates as a monetary policy tool for managing inflation in developing economies [1]. While some argue for its universal effectiveness, others suggest it may be less potent in developing economies [2]. Proponents of universal effectiveness cite its successful application in numerous developed economies to combat inflation [2,3]. They posit that the basic principles of economics are universally applicable, indicating that interest rate policy should perform as effectively in developing economies as it does in their more advanced counterparts.

Those economists who contend that interest rate policy is less effective in developing economies emphasize several factors that can impede its implementation and effectiveness in these countries [2,4]. For example, the prevalence of sizable informal sectors in developing economies, which often operate with distinct financial regulations compared to the formal sector. This circumstance can pose a challenge for central banks in effectively transmitting monetary policy signals to the entire economy [5]. The relatively weaker financial systems in developing economies can also impede the effective implementation of monetary policy [2]. Moreover, developing economies often grapple with substantial external debt, exposing them to fluctuations in global financial markets. This susceptibility can create difficulties for central banks as they endeavor to oversee and stabilize domestic inflation rates [6,7]. Furthermore, in developing economies, reduced central bank credibility leads expectations to be anchored in historical data, thereby increasing their reliance on past information. Consequently, this can lead to higher inflation by diminishing the efficacy of inflation targets as the primary influencers of inflation expectations [8].

Supply shocks can trigger substantial and enduring fluctuations in headline inflation. This can add complexity to the trade-off between economic output and inflation [9], posing challenges for monetary authorities and rendering monetary policy less effective in developing economies. In a high inflation scenario, understanding the implications of interest rates becomes a complex task. Assessing the true level of the real interest rate is particularly challenging when inflation expectations are in constant flux [10]. Further, in the presence of supply shocks, applying monetary tightening has a marked and positive influence on inflation in both advanced and emerging economies [11]. Rodrik and Velasco [12] argue that the sensitivity of investment and consumption to interest rates in developing economies can be low, limiting the effectiveness of interest rate policies in controlling inflation.

Developing economies face challenges in achieving consistent economic growth and have increasingly focused on using interest rates to control inflation, aiming for price stability in monetary policy. However, the relationship between inflation and interest rates remains unclear, with studies reporting varying causality directions. Some studies find unidirectional causality from interest rates to inflation [13,14], and inflation to interest rate [15–17], while others identify bidirectional relationships [18,19].

The debate about the effectiveness of interest rate policy to control inflation in developing economies is likely to endure. The evidence indicates that the effectiveness of such policies can vary due to structural and supply-side factors. Additionally, the literature does not provide a clear consensus on the causal relationship between inflation and interest rates. Nevertheless, it is important to recognize that interest rate policies remain a prominent strategy in many developing economies. This leads to the question: Why do central banks in these economies continue to favor interest rate-based monetary policy? To address this, our study utilizes wavelet transformation to dissect interest rate and inflation data across different frequency scales. One of the key advantages of wavelets lies in their ability to unveil concealed cyclic trends, patterns, and non-stationarity prevalent in economic time series data that may not be apparent with traditional time series analysis [20]. This innovative approach provides an opportunity for a meticulous exploration of the intricate causal interplay between these two pivotal macroeconomic variables. Conventional literature on causality often confines itself to the delineation of short- and long-run timescales. In contrast, our study advances the argument that numerous unexplored time and frequency scales exist between these two extremes [21], potentially exerting a profound impact on the causal relationship and its directional flow. It expands the horizons of the existing literature on causality and opens new avenues for understanding the nuances of monetary policy in developing economies.

## 2. Literature review

### 2.1. Theoretical framework

The theoretical framework of using interest rates as a monetary policy tool to control inflation is primarily rooted in the Quantity Theory of Money (QTM) and the Phillips Curve. The Quantity Theory of Money posits that a change in the rate of money supply growth leads to a corresponding change in the rate of growth in nominal income and in inflation [22]. Raising interest rates results in higher borrowing costs, prompting consumers and businesses to typically cut back on their spending and borrowing. This decrease in spending leads to a reduction in the demand for money which theoretically leads to lower inflation.

The Phillips Curve delineates an inverse correlation between inflation and unemployment [23], thereby signifying a trade-off for central banks. When central banks opt to increase interest rates, their objective is to mitigate inflation within an overheated economy. However, such a decision may entail a temporary elevation in unemployment levels. Conversely, the reduction of interest rates can catalyse economic activity but concurrently has the potential to exacerbate inflation. Consequently, central banks employ interest rates as a mechanism to attain equilibrium between inflation and employment.

### 2.2. Empirical debate

Monetary policy stands as a pivotal instrument for fostering economic growth in developing nations. The discourse surrounding this subject is long-lasting, with a multitude of studies elucidating its influence and efficacy. It holds the potential to achieve price stability, stimulate investments, and bolster economic vitality. Yet, the effectiveness of monetary policy in these contexts remains intricate and subject to debate. In this literature review, we amalgamate insights from pertinent research to illuminate the intricate connection between monetary policy and inflation in developing countries.

Inflation targeting is a monetary policy framework wherein the central bank defines a precise inflation target and deploys its policy tools to achieve this goal. Literature has demonstrated the efficacy of inflation targeting in reducing inflation rates and stimulating economic growth in developed countries. Notably, Masson et al. [2] observed that developing economies implementing inflation targeting experienced lower inflation compared to countries without such a policy framework. Nonetheless, arriving at a definitive conclusion is not as straightforward when considering developing economies [4] as the preconditions for adopting such a framework are not yet present in these economies [2]. In these contexts, the implementation and effectiveness of interest rate-based monetary policy can be hindered by structural and supply-side factors.

Alberola & Urrutia [5] argue that monetary policy actions are sacrificed due to the presence of an informal sector in developing economies. Their results indicate that informality can dampen inflation volatility in response to various shocks but, at the same time, diminish the efficacy of monetary policy. The monetary policy impact takes a longer time in relatively weaker financial systems in developing economies [24] as compared to developed financial systems. Further, when people have less trust in the central bank, they are more likely to base their expectations about future inflation on past inflation rates. Inflation targets by the central banks become less effective in impacting future inflation expectations [8].

According to Coletti et. al. [9], supply shocks can cause large and long-lasting fluctuations in inflation, which can make it difficult for central banks to manage inflation and economic output. This is especially true in developing economies, where monetary policy may be less effective. Further, in the presence of supply shocks, applying monetary tightening has a marked and positive influence on inflation in both advanced and emerging economies [11]. Under high inflation, it can be difficult to understand the effects of interest rates as the real interest rates can change quickly when inflation expectations are unstable [10]. Thus, the effectiveness of interest rate policies in controlling inflation may be limited in developing economies, because investment and consumption may be less sensitive to interest rates [12].

Thus, developing economies have been struggling to achieve steady economic growth [25]. Controlling inflation with the help of interest rates has become the only goal of many Central Banks during the previous few decades [26] because inflation targeting is a complete monetary framework that ensures price stability along with other monetary objectives [27–29]. However, inflationary pressures can lead to contractionary monetary policy that impacts economic growth, poverty, and inequality [30–33]. Thus, the direction of causality between inflation and interest rate is the prime concern because it is related to the potency of the monetary policy framework.

### 2.3. Causality debate

The literature does not provide any clarity on the direction of causality between the interest rate and inflation. Mehregan et al., [13] examine the causal relationship between inflation and interest rate by utilizing panel data from 24 developing countries. A unidirectional relationship from interest rate to inflation is established for 23 countries. Ahmadi et al.[14] rely on Hsiao’s causality test to establish the causal relationship from interest rate to inflation only for Qatar and Djibouti using quarterly data for sixteen Middle East-North Africa (MENA) countries for the period 1997–2008. No causality is found for the rest of the member countries. The Toda-Yamamoto causality test shows a unidirectional causal relationship from interest rate to inflation for the United Kingdom and Switzerland while for Germany the causality runs two-way [18]. Another study conducted on Turkey using monthly data shows a unidirectional causal relationship from inflation to interest rate between 2005(04)-2006(05) and from interest rate to inflation for 2015–2016 [34]. Asgharpur et al., [35] employ a panel causality approach to 40 Islamic countries to explore the causal nexus between interest rate and inflation. The study establishes the unidirectional relationship from interest rate to inflation. Mirza & Rashidi [36] consider two scenarios for assessing the causal relationship between the variables: lending rate & inflation and real interest rate and inflation. The study utilizes panel data ranging from 2006 to 2013 for South Asian Association for Regional Cooperation (SAARC) countries and finds no causal relationship for the first scenario and a bidirectional causal relationship for the second scenario.

Fatima & Sahibzada [19] determine the nature of the relationship between inflation, interest rate, and money supply for the economy of Pakistan from 1980–2010. The Granger causality analysis indicates the existence of a bidirectional relationship between inflation and interest rate which corroborates with the findings in [33]. Nezhad & Zarea [37] examine the causality relationship between inflation and interest rate in Iran. Toda and Yamamoto’s Granger causality analysis shows one-way causality from the interest rate to the inflation rate. Amaefula [38] finds strong evidence of unidirectional Granger causality from interest rate to inflation rate for Nigeria using monthly data from 1995 to 2014 whereas Alimi & Ofonyelu [17] employs Toda-Yamamoto test to establish a unidirectional relationship from inflation to nominal interest rate for Nigeria using data for the period 1970–2011 which is in line with the findings for Kenya [39], Jorden [40], Pakistan [15], and Bangladesh [16].

Based on the synopsis of previous studies above, there is a lack of consensus among researchers concerning the efficacy of interest rate policies in managing inflation within developing economies due to the structural and supply-side factors. Furthermore, the direction of causality between interest rates and inflation in developing nations remains a subject of debate. The sample size, econometric methods, frequency, and time scales could be the possible reasons behind the conflicting causality results. Literature covers the first two reasons, sample size and econometric methods well [41–44] however, the frequency and time scale impact has not been considered. Literature on causality considers only the short- and long-run as time scales while this study argues that there exist more time and frequency scales in between the short- and long-run that may impact the causal relationship and its direction. Therefore, we employ the wavelet transformation to decompose the interest rate and inflation series into different frequency scales. This will help us to study the causal dynamics between the two important macroeconomic variables in detail.

## 3. Data & methodology

This study utilizes international financial statistics (IFS) and central banks’ repositories to collect the data on Consumer Price Index (CPI) based inflation and Policy Rate (PR) of twenty-two developing countries for varying periods (see Table 2, Col 1, 2). This study aims to investigate the causal relationship between inflation and interest rates through a time-frequency analysis, necessitating the use of high-frequency data. The selection of the countries in our sample is contingent on the accessibility of monthly data concerning these macroeconomic variables. Time series analysis of macroeconomic indicators is generally grounded in the time domain and ignores the frequency domain. Spectrum analysis allows us to decompose the series into a spectrum of cycles of varying length that helps to extract the main oscillatory components of the series [45,46] including low frequency (trend), medium frequency (cycles), and high frequency (noise) components. There are different tools available to transform the time series into these components including the Fourier and Wavelet transformations. The Fourier transformation requires the stationarity of the data [47] whereas the macroeconomic variables are less likely to be integrated of order zero (i.e., stationary). To explore the scale-dependent causal linkages between inflation and policy rate, we employ Wavelet transformation to decompose the series into various frequency scales. Wavelet transformation has an edge over the Fourier transformation as its window size adjusts optimally both for low and high frequencies [46]. Further, its good frequency and time resolutions enable it to capture movements both at low and high frequencies which allows it to cater for outliers, regime changes, and shocks [48]. For wavelet decomposition, we need to select a suitable filter like Haar and Daubechies. The Haar filter is simple but neither continuous nor differentiable however the Daubechies is an orthogonal, smooth, symmetric, and localized filter [48]. Therefore, this study uses the Daubechies’s Daub4 wavelet filter while decomposing the series.

After decomposing the inflation and policy rate series for all the selected countries, we estimate correlation coefficients for inflation and policy rate at different scales to understand the direction and strength of the relationship between the variables. The t-test is used to establish the statistical significance of the correlation coefficients. To explore the direction of causality, we employ the Granger Causality test to the pair of variables at various scales. The direction of the relationship and causality together would help us answer the question of whether the policy rate is an appropriate tool for controlling inflation.

### 3.1. Wavelet decomposition

This study employs Daubechies’s wavelet to decompose inflation and policy rate and considers the length of the business cycle to sixty-four months [46]. The series is decomposed into different frequency components of length between (2^*k*^, 2^*k*+1^) months where *k* = 1,2,3,…,5 keeping in view the monthly frequency of our data [49,50]. Both series are decomposed into five orthogonal frequency components, trends, and business cycles (Table 1). To better understand the decomposition, following Hanif et al., [48] & Tiwari et al., [51], the components are categorized as noise (D1), cycles (D2-D5), business cycle, and trend (Col 3, Table 1).

**Table 1:** Frequency scales.

Scales	Frequency	Category
D1	2–4 Months	Noise
D2	4–8 Months	Cycles
D3	8–16 Months	
D4	16–32 Months	
D5	32–64 Months	
Business Cycle	4–64 Months	Business Cycle
Trend	More than 64 Months	Trend

**Fig 1:**
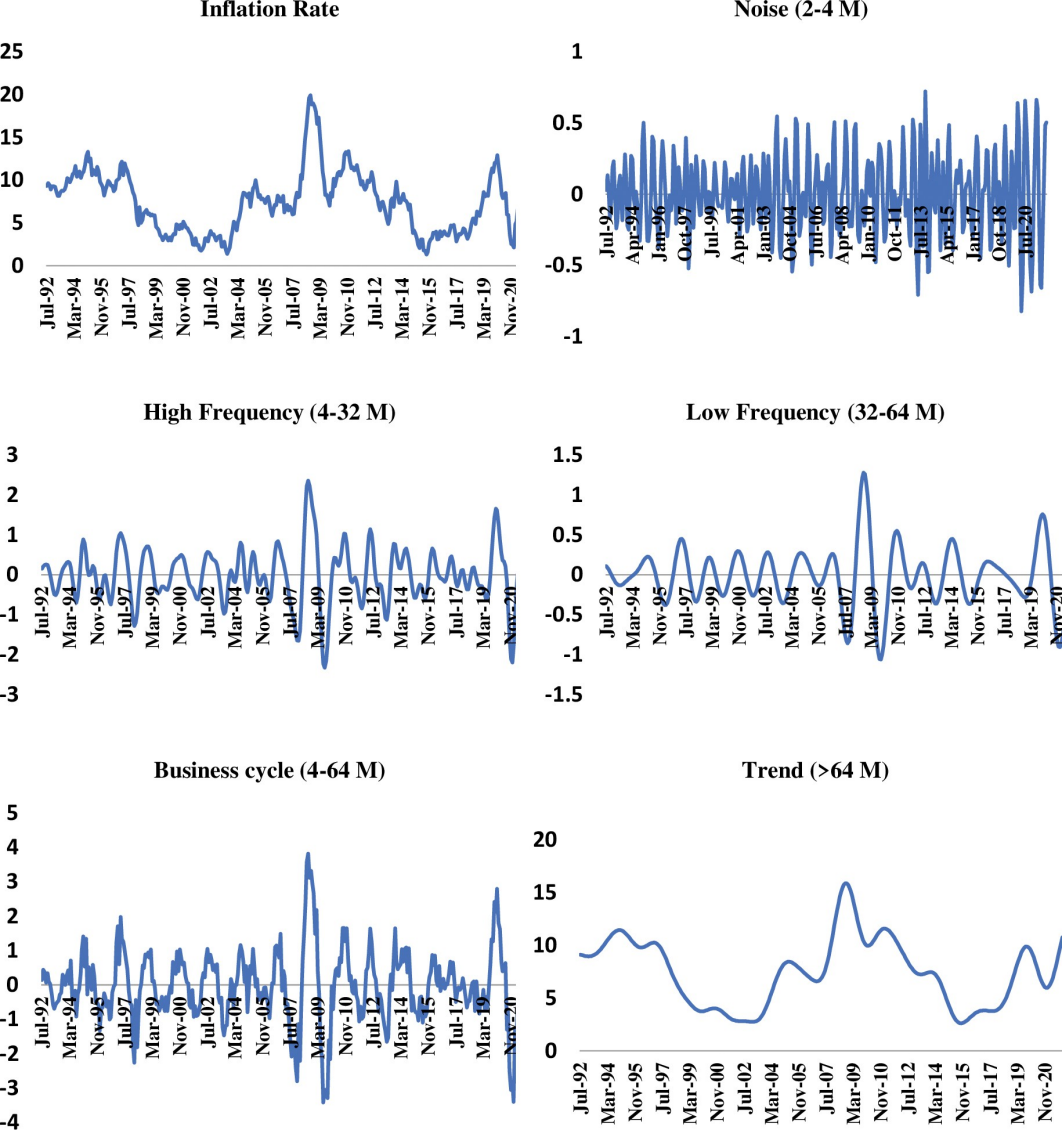
Wavelet decomposition (Inflation, Pakistan).

These frequency scales depict varying cyclical fluctuations which are usually ignored while conducting the conventional causal analysis. The implication of ignoring these cyclic fluctuations is that we could not study the relationship between the variables at different scales that exist between the short- and long-run. Fig 1 indicates the phenomenon: cyclical fluctuations recede as the aggregation increases over the months.

## 4. Results & discussion

**Fig 2:**
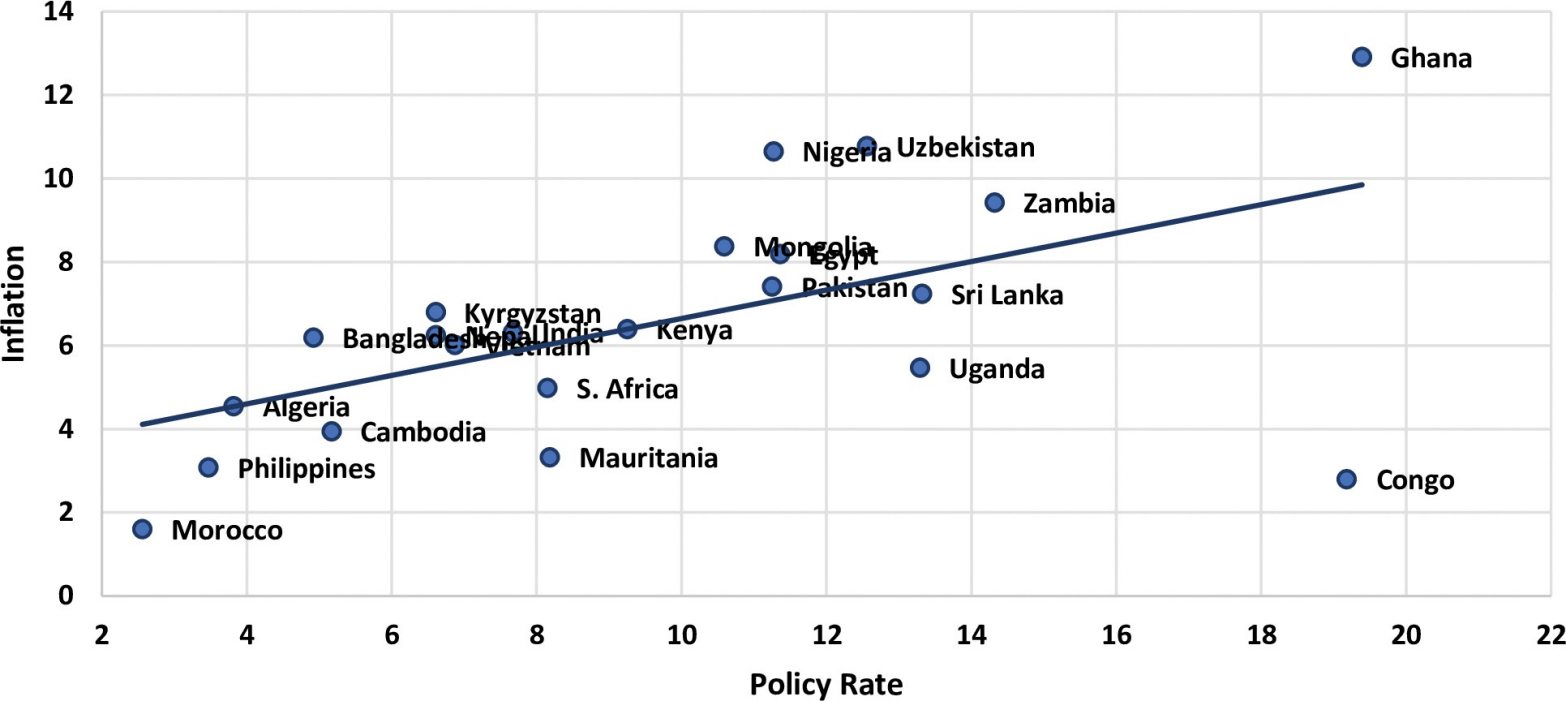
Average inflation and policy rates.

Table 2 below provides descriptive statistics on the inflation and policy rates of the selected twenty-two developing countries. Theoretically speaking, the higher the interest rate, the lower the inflation rate like in the case of Congo, the average policy rate is at 19.18 and the average inflation rate is at 2.80. Whereas the mean inflation and policy rates for other countries like Morocco, Ghana, Nigeria, Uzbekistan, etc. do not follow the inverse relationship as per the theory. On balance, Fig 2 shows a positive relationship between the two rates i.e., the higher the policy rate, the higher the inflation. These initial findings raise a valid question on the appropriateness of the policy rate as a monetary tool and the disagreement among the researchers regarding the direction of causality motivates us to explore the more time and frequency scales that exist between the short- and long-run.

**Table 2:** Descriptive statistics.

Country	Period	Mean	Maximum	Minimum	St. Dev	Skewness	Kurtosis	JB Test	Prob	Obs.
	**Inflation**									
Pakistan	1992(7)-2022(12)	7.42	19.89	1.346	3.52	0.64	3.72	33.78	0.00	366
India	1992(7)-2022(12)	6.32	16.88	0.42	2.74	0.63	3.26	25.37	0.00	366
Kyrgyzstan	2004(1)-2022(12)	6.80	24.64	-0.76	5.73	1.07	3.45	45.93	0.00	228
Algeria	2006(1)-2022(12)	4.55	9.64	-0.33	2.22	0.21	2.49	3.80	0.14	204
Congo	2006(2)-2022(12)	2.80	10.61	-4.39	2.64	0.56	3.57	13.51	0.00	203
Morocco	2008(1)-2022(12)	1.61	7.88	-1.96	1.69	1.63	6.54	173.86	0.00	180
Vietnam	2002(12)-2022(12)	6.02	22.20	-0.81	4.71	1.52	5.14	139.82	0.00	241
Bangladesh	2011(1)-2022(12)	6.19	11.45	4.80	1.50	1.97	6.37	161.86	0.00	144
Mongolia	2007(1)-2022(12)	8.38	25.68	-0.27	5.23	0.95	4.40	44.79	0.00	192
Nepal	2006(1)-2022(12)	6.25	12.13	-2.31	3.09	-0.54	2.83	10.26	0.00	204
Sri Lanka	2011(1)-2022(12)	7.24	42.15	-0.59	8.29	3.14	12.40	768.19	0.00	144
Cambodia	2002(1)-2022(12)	3.95	26.62	-6.71	4.46	2.47	12.25	1156.80	0.00	252
Egypt	1993(7)-2022(12)	8.19	25.21	1.78	4.71	1.10	4.55	108.18	0.00	354
Ghana	1998(9)-2022(12)	12.92	38.80	0.43	6.90	1.83	6.95	353.64	0.00	292
Kenya	2010(2)-2022(12)	6.40	16.97	3.18	2.79	1.93	6.99	200.10	0.00	155
Mauritania	2011(1)-2022(12)	3.33	9.55	-0.67	1.64	0.68	4.21	20.01	0.00	144
Nigeria	2006(1)-2022(12)	10.65	17.84	2.93	3.03	0.01	2.63	1.10	0.57	204
S. Africa	1998(3)-2022(12)	4.99	12.43	-2.01	2.36	-0.15	4.37	24.75	0.00	298
Philippines	2011(1)-2022(12)	3.08	7.71	-0.52	1.58	0.35	3.32	3.65	0.16	144
Uzbekistan	2011(1)-2022(12)	10.78	16.55	6.67	2.38	0.39	2.54	5.09	0.07	144
Uganda	2011(1)-2022(12)	5.47	21.54	1.52	4.35	2.06	6.91	194.47	0.00	144
Zambia	2011(1)-2022(12)	9.43	19.51	5.47	4.26	1.15	2.83	32.25	0.00	144
**Policy Rate**										
Pakistan	1992(7)-2022(12)	11.25	20.20	6.06	3.47	0.55	2.49	22.72	0.00	366
India	1992(7)-2022(12)	7.67	12.21	3.94	2.30	0.59	2.64	23.46	0.00	366
Kyrgyzstan	2004(1)-2022(12)	6.61	16.04	0.84	3.27	1.02	3.31	40.61	0.00	228
Algeria	2006(1)-2022(12)	3.82	4.03	2.93	0.30	-1.89	5.52	177.09	0.00	204
Congo	2006(2)-2022(12)	19.18	74.93	1.82	17.32	1.41	4.67	91.75	0.00	203
Morocco	2008(1)-2022(12)	2.56	3.48	1.46	0.63	-0.37	1.90	13.30	0.00	180
Vietnam	2002(12)-2022(12)	6.87	15.10	3.90	2.61	1.71	5.55	183.82	0.00	241
Bangladesh	2011(1)-2022(12)	4.92	5.81	3.96	0.31	-1.41	7.62	176.58	0.00	144
Mongolia	2007(1)-2022(12)	10.59	15.03	5.66	2.17	-0.59	2.98	11.14	0.00	192
Nepal	2006(1)-2022(12)	6.61	8.04	4.93	0.91	-0.26	2.51	4.40	0.11	204
Sri Lanka	2011(1)-2022(12)	13.32	15.79	4.25	3.66	-1.81	4.41	90.79	0.00	144
Cambodia	2002(1)-2022(12)	5.17	10.15	0.62	2.22	0.22	2.57	4.00	0.13	252
Egypt	1993(7)-2022(12)	11.36	19.37	8.04	2.70	1.06	3.34	68.14	0.00	354
Ghana	1998(9)-2022(12)	19.39	41.71	12.37	5.61	0.90	3.60	44.53	0.00	292
Kenya	2010(2)-2022(12)	9.25	18.58	5.52	2.71	1.74	6.31	149.99	0.00	155
Mauritania	2011(1)-2022(12)	8.18	9.10	6.91	0.98	-0.36	1.14	23.97	0.00	144
Nigeria	2006(1)-2022(12)	11.27	15.55	5.86	2.42	-0.75	2.75	19.77	0.00	204
S. Africa	1998(3)-2022(12)	8.15	21.98	3.35	3.62	1.37	5.10	148.93	0.00	298
Philippines	2011(1)-2022(12)	3.47	5.08	1.92	0.81	-0.44	2.28	7.85	0.01	144
Uzbekistan	2011(1)-2022(12)	12.56	17.04	8.77	2.47	-0.09	1.78	9.15	0.01	144
Uganda	2011(1)-2022(12)	13.29	28.39	6.39	5.61	0.83	3.04	16.78	0.00	144
Zambia	2011(1)-2022(12)	14.32	22.45	7.29	3.88	0.21	1.97	7.38	0.02	144

This study utilizes correlation and causality analysis to test the appropriateness of the policy rate as a monetary tool. Theoretically, we should expect a negative and significant correlation between the policy rate and inflation with the direction of causality running from policy rate to inflation. Correlation and causality results are furnished in Tables 3 and 4 respectively.

**Table 3:** Correlation between inflation and policy rates.

	Policy Rate																							
	Levels			Noise (D1)			D2			D3			D4			D5			Business Cycle			Trend		
Country	Level	L1	L2	Level	L1	L2	Level	L1	L2	Level	L1	L2	Level	L1	L2	Level	L1	L2	Level	L1	L2	Level	L1	L2
Pakistan	+ve*	+ve*	+ve*	-ve*	+ve*	+ve	+ve*	+ve*	+ve	+ve*	+ve*	+ve*	+ve*	+ve*	+ve*	+ve*	+ve*	+ve*	+ve*	+ve*	+ve*	+ve*	+ve*	+ve*
India	+ve*	+ve*	+ve*	+ve*	+ve*	-ve	+ve*	+ve*	+ve	+ve*	+ve*	+ve	+ve*	+ve*	+ve	+ve*	+ve*	+ve*	+ve*	+ve*	+ve	+ve*	+ve*	+ve*
Kyrgyzstan	+ve*	+ve*	+ve*	+ve*	-ve	-ve	+ve*	-ve	-ve*	+ve*	+ve	+ve	+ve	+ve	+ve*	+ve*	+ve	+ve*	+ve	+ve	+ve*	+ve*	+ve*	+ve*
Algeria	-ve*	-ve*	-ve*	+ve*	+ve	-ve	+ve*	+ve	+ve*	-ve	-ve	-ve*	-ve*	-ve*	-ve*	-ve*	-ve*	-ve*	-ve*	-ve*	-ve*	-ve*	-ve*	-ve*
Congo	+ve	+ve	+ve*	-ve*	+ve	+ve*	-ve*	-ve*	+ve*	-ve*	-ve*	-ve*	-ve*	-ve	+ve*	+ve*	+ve*	+ve*	-ve	-ve*	+ve	+ve	+ve*	+ve*
Morocco	-ve*	-ve*	-ve*	+ve*	+ve	+ve	-ve	-ve*	+ve*	-ve	+ve*	+ve	-ve	-ve*	+ve*	-ve*	-ve*	-ve*	-ve*	-ve*	+ve*	-ve*	-ve*	-ve*
Vietnam	+ve*	+ve*	+ve*	+ve*	+ve*	-ve*	+ve*	+ve*	+ve*	+ve*	+ve*	+ve*	+ve*	+ve*	+ve*	+ve*	+ve*	+ve*	+ve*	+ve*	+ve*	+ve*	+ve*	+ve*
Bangladesh	+ve*	+ve*	+ve*	+ve	+ve*	+ve	+ve	+ve*	+ve*	+ve	+ve	+ve	-ve*	+ve*	+ve*	+ve*	-ve*	-ve*	+ve*	+ve	+ve	+ve*	+ve*	+ve*
Mongolia	-ve*	-ve*	-ve*	+ve*	+ve	+ve	+ve	+ve*	+ve*	+ve*	+ve	+ve	+ve	+ve	+ve	-ve*	+ve*	+ve*	+ve	+ve	+ve	-ve*	-ve*	-ve*
Nepal	+ve*	+ve*	+ve*	+ve*	+ve*	+ve	+ve*	+ve*	+ve*	+ve*	+ve*	+ve*	+ve*	+ve*	+ve*	+ve	+ve	+ve	+ve*	+ve*	+ve*	+ve*	+ve*	+ve*
Sri Lanka	-ve*	-ve*	-ve*	+ve*	+ve*	-ve*	+ve*	+ve*	+ve	+ve*	+ve*	+ve*	+ve*	+ve*	+ve*	+ve	+ve	+ve	+ve*	+ve*	+ve*	-ve*	-ve*	-ve
Cambodia	+ve*	+ve*	+ve*	-ve	+ve	+ve	+ve	+ve*	+ve*	+ve	+ve*	+ve*	+ve*	+ve*	+ve*	+ve*	+ve*	+ve*	+ve	+ve*	+ve*	+ve*	+ve*	+ve*
Egypt	+ve*	+ve*	+ve*	+ve	-ve*	-ve*	+ve*	+ve*	+ve*	+ve*	+ve*	+ve*	+ve*	+ve*	+ve*	+ve*	+ve*	+ve*	+ve*	+ve*	+ve*	+ve*	+ve*	+ve*
Ghana	+ve*	+ve*	+ve*	+ve*	+ve*	-ve	+ve*	+ve*	-ve	+ve*	+ve*	+ve*	+ve*	+ve*	+ve*	+ve*	+ve*	+ve*	+ve*	+ve*	+ve*	+ve*	+ve*	+ve*
Kenya	+ve*	+ve*	+ve*	+ve	+ve	+ve*	+ve	+ve	+ve	+ve*	+ve*	+ve*	+ve*	+ve*	+ve*	+ve*	+ve*	+ve*	+ve*	+ve*	+ve*	+ve*	+ve*	+ve*
Mauritania	+ve	+ve	+ve	-ve*	-ve	-ve	-ve*	-ve*	-ve*	-ve*	-ve*	-ve*	-ve*	-ve*	-ve*	-ve*	+ve*	+ve	-ve*	-ve*	-ve	+ve*	+ve*	+ve*
Nigeria	+ve*	+ve*	+ve*	-ve	-ve	-ve	+ve	+ve	+ve	+ve*	+ve*	+ve*	+ve*	+ve*	+ve*	+ve*	+ve*	+ve*	+ve*	+ve*	+ve*	+ve*	+ve*	+ve*
S. Africa	+ve*	+ve*	+ve*	+ve*	-ve	-ve*	+ve*	+ve	-ve*	+ve*	+ve*	+ve*	+ve*	+ve*	+ve*	+ve*	+ve*	+ve*	+ve*	+ve*	+ve*	+ve*	+ve*	+ve*
Philippines	+ve*	+ve	+ve*	+ve*	+ve*	+ve	+ve*	+ve*	+ve*	+ve*	+ve*	+ve*	+ve*	+ve*	+ve*	+ve	+ve*	+ve*	+ve*	+ve*	+ve*	-ve*	+ve*	+ve*
Uzbekistan	+ve*	+ve	+ve*	+ve*	+ve	-ve*	+ve*	+ve*	+ve	+ve*	+ve*	+ve	+ve*	+ve*	+ve	+ve	+ve*	+ve*	+ve*	+ve*	+ve	+ve*	+ve*	+ve*
Uganda	+ve*	+ve	+ve*	-ve*	+ve*	+ve*	+ve*	+ve*	+ve*	+ve*	+ve*	+ve*	+ve*	+ve*	+ve*	+ve*	+ve*	+ve*	+ve*	+ve*	+ve*	+ve*	+ve*	+ve*
Zambia	+ve*	+ve*	+ve*	-ve*	+ve*	+ve*	+ve	+ve*	+ve*	+ve*	+ve*	+ve*	+ve*	+ve*	+ve*	+ve*	+ve*	+ve*	+ve*	+ve*	+ve*	+ve*	+ve*	+ve*

**Table 4:** Causality analysis.

	Policy Rate																							
	Levels			Noise (D1)			D2			D3			D4			D5			Business Cycle			Trend		
Country	Level	L1	L2	Level	L1	L2	Level	L1	L2	Level	L1	L2	Level	L1	L2	Level	L1	L2	Level	L1	L2	Level	L1	L2
Pakistan	↔	→	→	No	No	No	←	→	→	↔	→	↔	←	→	→	↔	↔	↔	↔	No	No	↔	↔	↔
India	No	No	No	→	→	↔	→	→	→	→	→	→	↔	↔	↔	↔	↔	↔	→	→	No	↔	↔	↔
Kyrgyzstan	No	No	No	↔	→	↔	↔	←	←	↔	↔	↔	↔	↔	↔	↔	↔	↔	→	→	→	↔	↔	↔
Algeria	→	No	No	No	No	No	↔	↔	←	No	No	No	↔	←	↔	↔	↔	↔	No	No	No	↔	↔	↔
Congo	↔	No	No	No	No	No	←	←	←	↔	↔	↔	↔	↔	↔	↔	↔	↔	←	←	←	↔	↔	↔
Morocco	No	No	No	No	→	No	No	No	No	No	No	No	↔	↔	↔	↔	↔	↔	No	No	No	↔	↔	↔
Vietnam	↔	←	↔	↔	←	↔	↔	↔	↔	↔	↔	↔	↔	↔	↔	↔	↔	↔	↔	↔	↔	↔	↔	↔
Bangladesh	No	No	No	→	→	→	→	↔	←	←	No	→	→	→	→	↔	↔	↔	→	→	→	↔	↔	↔
Mongolia	No	No	No	No	No	No	→	→	→	No	No	No	→	↔	↔	↔	↔	↔	No	No	No	↔	↔	↔
Nepal	No	No	No	No	No	No	No	No	No	No	No	No	No	No	No	→	→	↔	No	No	No	↔	↔	↔
Sri Lanka	→	→	→	→	→	→	→	→	↔	↔	↔	↔	↔	↔	↔	↔	↔	↔	→	→	→	↔	↔	↔
Cambodia	←	←	←	←	←	←	No	→	←	←	↔	↔	↔	↔	↔	↔	↔	↔	←	←	←	↔	↔	↔
Egypt	↔	↔	↔	↔	↔	↔	↔	↔	↔	↔	↔	↔	←	↔	↔	↔	↔	↔	→	→	→	↔	↔	↔
Ghana	↔	↔	↔	←	←	←	←	↔	↔	←	↔	↔	↔	↔	↔	↔	↔	↔	←	←	←	↔	↔	↔
Kenya	↔	↔	↔	No	No	No	↔	↔	↔	↔	↔	↔	↔	↔	↔	↔	↔	↔	←	←	←	↔	↔	↔
Mauritania	No	No	No	No	No	←	←	←	←	No	No	No	No	No	No	↔	↔	↔	No	No	No	↔	↔	↔
Nigeria	No	↔	↔	↔	↔	↔	↔	↔	No	↔	→	→	←	↔	↔	↔	↔	↔	←	←	→	↔	↔	→
S. Africa	→	↔	→	→	↔	→	↔	↔	↔	↔	↔	↔	↔	↔	→	↔	↔	↔	↔	↔	↔	↔	↔	↔
Philippines	↔	↔	→	No	No	→	→	→	→	↔	↔	←	↔	↔	↔	↔	↔	↔	No	No	No	↔	↔	↔
Uzbekistan	←	No	→	→	→	→	↔	←	→	↔	↔	↔	↔	↔	↔	↔	↔	↔	No	No	No	↔	↔	↔
Uganda	←	↔	→	↔	↔	↔	↔	↔	←	↔	↔	↔	↔	↔	↔	↔	↔	No	→	→	→	↔	↔	↔
Zambia	←	←	←	↔	↔	↔	←	←	←	←	↔	↔	↔	↔	↔	↔	↔	↔	←	←	←	↔	↔	↔

↔, →, ← imply causality runs two-ways, from policy rate to inflation and from inflation to policy rate respectively.

In Table 3, we observed a negative and significant correlation between the policy rate and inflation, at levels, for four countries including Algeria, Morocco, Mongolia, and Sri Lanka which is in line with the theory. However, interestingly, no causal relationship is observed for these countries [14] except for Sri Lanka (Table 4). Policy rate causes inflation in Sri Lanka with a negative and significant relationship [13] which contradicts the finding in [36] of bidirectional causality for the SAARC countries. At levels, no causality in case of India and the bidirectional causality in the case of Pakistan [19] might lead [36] to conclude the bidirectional causality for the panel of SAARC countries. Positive and significant correlation is observed for the remaining eighteen countries with mixed results on the direction of causality (Table 3) which is not in line with the theory. Unidirectional causality runs from policy rate to inflation for Pakistan, Sri Lanka, and South Africa whereas causality runs from inflation to policy rate for Cambodia and Zambia. Two-way relationship is established for Egypt, Ghana, Kenya, and Nigeria.

Considering the noise series (D1), a negative and significant correlation is recorded for five countries at level whereas lagged policy rate has a significant and positive association with inflation (Table 3). Three out of these five countries (Pakistan, Congo, Mauritania) show no causal relationship at level for the noise series and the remaining two countries (Uganda & Zambia) show a two-way causal relationship (Table 4). The direction of causality runs from lagged policy rate to inflation in the case of Bangladesh, Sri Lanka, and Uzbekistan with a positive correlation whereas inflation causes the lagged policy rate in the case of Cambodia and Ghana.

For D2 (4–8 months) component at level, negative and significant correlation is observed for only Congo and Mauritania where inflation causes the policy rate. For the remaining countries, positive and significant correlation contradicts the theory while the causality runs from policy rate to inflation for five countries, from inflation to policy rate for five countries, and a bidirectional relationship is established for nine countries. The lagged policy rate causes inflation only in the case of India but with a positive and significant correlation meaning that a positive shock to the policy rate would lead to higher inflation in the future. On balance, the findings for the D2 component reject the policy rate as an appropriate monetary tool.

For D3 (8–16 months), no causality is observed for Algeria, Morocco, Mongolia, Nepal, and Mauritania [14]. Congo is the only country that has a significant and negative correlation with bidirectional causality. Thirteen other countries are showing bidirectional causality but with positive and significant correlation which is not in line with our hypothesis: there is a negative relationship between the policy rate and inflation. Policy rate causes inflation in Nigeria [38] with the positive direction of the relationship between the variables.

While considering D4 (16–32 months), a lagged policy rate causes inflation in the case of Pakistan and Bangladesh with a positive and significant correlation between the variables of interest. On balance, the remaining countries exhibit a two-way causality while the negative and significant correlation is only observed for Algeria and Mauritania. It is pertinent to mention that no causal relationship is observed for Mauritania. No causality may be attributed to the existence of a parallel financial market in Mauritania [52].

For D5 (32–64 months), results indicate a bidirectional causal relationship between the policy rate and inflation for all the selected developing countries however negative correlation is found only for Algeria and Morocco. Positive and significant correlation for the rest of the economies is not in line with our theoretical hypothesis.

For the business cycle (4–64 months) series, policy rate causes inflation in the case of India, Kyrgyzstan, Bangladesh, Sri Lanka, Egypt, and Uganda however the correlation is positive and significant between the variables of interest. This means an increase in interest rate would further add fuel to inflation. While Congo, Cambodia, Ghana, Kenya, Nigeria, and Zambia are experiencing a reverse causality i.e., inflation is causing the policy rate with a significant positive correlation except for Congo meaning that an increase in inflation would push the interest rate in the upward direction. These results corroborate the findings in the literature for countries like Nigeria and Kenya [17,39].

Considering the trend (> 64 months), a negative and significant relationship is established only for Algeria, Morocco, Mongolia, and Sri Lanka while the rest of the countries show a positive and significant correlation between the policy rate and inflation. The direction of causality is unanimously bidirectional for all the selected developing countries.

To sum up, Algeria, Morocco, and Mauritania are the countries bearing a negative and significant relationship between the policy rate and inflation for most of the frequency scales. However, these countries exhibit either no causality or bidirectional causality for most of the scenarios. Further, Mauritania experiences reverse causality (from inflation to policy rate) for the D2 component. For the rest of the countries, the correct combination of correlation sign and direction of causality is not found. Hence, we reject the hypothesis that increasing the policy rate would control the inflation in developing countries under consideration.

## 5. Conclusion

This study examines the suitability of policy rates as instruments for managing inflation in 22 developing countries using wavelet decomposition. Theoretically, the policy rate and inflation are negatively correlated i.e., the inflation rate would decline in response to an increase in policy rate and the direction of causality runs from policy rate to inflation. The investigation reveals several noteworthy findings at different time scales.

*At levels*, on average, a positive relationship exists between policy rate and inflation across the countries studied (Fig 2). The direction of causality is found to be both uni- and bi-directional with positive and significant correlations between these variables. The only exception is the Sri Lanka where the policy rate does Granger cause inflation with negative correlation which is in line with the theory.

The *short run* analysis is based on the two components of the series namely D1(2–4 months) and D2 (4–8 months). First component analysis reveals that the policy rate and inflation are negatively and significantly correlated with bidirectional causality for Uganda and Zambia only. Unidirectional causality runs from policy rate to inflation in the case of Bangladesh, Sri Lanka, and Uzbekistan with a positive and statistically significant correlation. In the context of second component the policy rate causes inflation in case of India, Philippines, and Mongolia with positive and statistically significant correlation. This suggests that an increase in the policy rate could potentially lead to a rise in inflation within these countries. While the causality direction remains ambiguous for the remaining countries in the study, it is important to note that there is a positive correlation between the policy rate and inflation.

The *medium short run* analysis utilizes the D3 (8–16 months), D4 (16–32 months), and D5 (32–64 months) components of the policy rate and inflation. For the first component (D3), Congo is the only country having a significant and negative correlation with bidirectional causality. Thirteen other countries are showing bidirectional causality but with positive and significant correlation. While considering the D4 & D5 components, on balance, a two-way causality with a positive correlation is found. In the *long run* (> 64 months), the direction of causality is unanimously bidirectional for all the selected developing countries with a positive and significant correlation between the policy rate and inflation.

Theory suggests that a negative causal relationship between the policy rate and inflation is instrumental for central banks to implement tight monetary policy as a measure to effectively control inflation. However, this exercise reveals that the developing economies bear a positive causal relationship between the policy rate and inflation. This indicates that as the central bank raises interest rates, it triggers an increase in price levels. Furthermore, in response to rising prices, the central bank continues to raise the policy rate, perpetuating this cycle. Given the observed positive causal relationship between the policy rate and inflation in developing economies, central banks should explore structural and supply-side elements to disrupt this cycle and successfully manage inflation.

## References

[1] Twinoburyo E. N. and Odhiambo N. M., “Monetary policy and economic growth: A review of international literature,” *Journal of Central Banking Theory and Practice*, vol. 7, no. 2. 2018. doi: 10.2478/jcbtp-2018-0015

[2] MASSON P. R., SAVASTANO M. A., and SHARMA S., “Can Inflation Targeting be a Framework for Monetary Policy in Developing Countries?,” *Finance Dev*, vol. 35, pp. 34–37, 1998.

[3] Mallick S. K. and Sousa R. M., “Real effects of monetary policy in large emerging economies,” *Macroecon Dyn*, vol. 16, no. SUPPL. 2, 2012, doi: 10.1017/S1365100511000319

[4] Laxton D. and Pesenti P., “Monetary rules for small, open, emerging economies,” *J Monet Econ*, vol. 50, no. 5, 2003, doi: 10.1016/S0304-3932(03)00057-6

[5] Alberola E. and Urrutia C., “Does informality facilitate inflation stability?,” *J Dev Econ*, vol. 146, 2020, doi: 10.1016/j.jdeveco.2020.102505

[6] Ampah I. K. and Kiss G. D., “Economic policy implications of external debt and capital flight in sub-Saharan Africa’s heavily indebted poor countries,” *Society and Economy*, vol. 41, no. 4, 2019, doi: 10.1556/204.2019.41.4.8

[7] Helmy H. E., “The external debt-inflation nexus in Egypt,” *J Public Aff*, vol. 22, no. S1, 2022, doi: 10.1002/pa.2802

[8] Güler A., “Does monetary policy credibility help in anchoring inflation expectations? Evidence from six inflation targeting emerging economies,” *Journal of Central Banking Theory and Practice*, vol. 10, no. 1, 2021, doi: 10.2478/jcbtp-2021-0005

[9] Coletti D., Lalonde R., Masson P., Muir D., and Snudden S., “Commodities and monetary policy: Implications for inflation and price level targeting,” *J Policy Model*, vol. 43, no. 5, 2021, doi: 10.1016/j.jpolmod.2021.02.013

[10] Taylor J. B., “Inflation targeting in high inflation emerging economies: Lessons about rules and instruments,” *Journal of Applied Economics*, vol. 22, no. 1. 2019. doi: 10.1080/15140326.2019.1565396

[11] Bhattacharya R. and Jain R., “Can monetary policy stabilise food inflation? Evidence from advanced and emerging economies,” *Econ Model*, vol. 89, 2020, doi: 10.1016/j.econmod.2019.10.005

[12] Rodrik D. and Velasco A., “Short-Term Capital Flows,” Cambridge, MA, Sep. 1999. doi: 10.3386/w7364

[13] Mehregan N., Ezati M., and Hossein A., “CAUSAL RELATIONSHIP BETWEEN THE INTEREST RATE AND INFLATION USING PANEL DATA,” 2006.

[14] Ahmadi M., Fallahi M., and Khosravi S., “Hsiao’s Causality Test between Interest Rate and Inflation Rate for Mena Countries Group,” *Economic Growth and Development Research*, 2011.

[15] Mahmood Ali T., Tariq Mahmood M., and Bashir T., “Impact of Interest Rate, Inflation and Money Supply on Exchange Rate Volatility in Pakistan,” *World Appl Sci J*, vol. 33, no. 4, 2015.

[16] Chowdhury S. A., Md Yousuf, Md. N Uddin, and M. J Uddin, “Nominal Interest Rate, Inflation Money and Market Link in Bangladesh: An Econometric Analysis,” *Asian Journal of Humanity*, *Art and Literature*, vol. 7, no. 1, 2020, doi: 10.18034/ajhal.v7i1.501

[17] Alimi S. R and Ofonyelu C. C, “TODA-YAMAMOTO CAUSALITY TEST BETWEEN MONEY MARKET INTEREST RATE AND EXPECTED INFLATION: THE FISHER HYPOTHESIS REVISITED,” 2013.

[18] Dritsaki C., “Toda-Yamamoto Causality Test between Inflation and Nominal Interest Rates: Evidence from Three Countries of Europe,” *International Journal of Economics and Financial Issues*, vol. 7, no. 6, 2017.

[19] Fatima N. and Sahibzada S. A., “Empirical evidence of fisher effect in Pakistan,” *World Appl Sci J*, vol. 18, no. 6, 2012, doi: 10.5829/idosi.wasj.2012.18.06.1118

[20] Adebayo T. S., Oladipupo S. D., Adeshola I., and Rjoub H., “Wavelet analysis of impact of renewable energy consumption and technological innovation on CO2 emissions: evidence from Portugal,” *Environmental Science and Pollution Research*, vol. 29, no. 16, 2022, doi: 10.1007/s11356-021-17708-8 34817815

[21] Adebayo T. S., Ağa M., and Kartal M. T., “Analyzing the co-movement between CO2 emissions and disaggregated nonrenewable and renewable energy consumption in BRICS: evidence through the lens of wavelet coherence,” *Environmental Science and Pollution Research*, vol. 30, no. 13, 2023, doi: 10.1007/s11356-022-24707-w 36588131

[22] Duck N. W., “Some International Evidence on the Quantity Theory of Money,” *J Money Credit Bank*, vol. 25, no. 1, 1993, doi: 10.2307/2077816

[23] Phillips A. W., “The Relation Between Unemployment and the Rate of Change of Money Wage Rates in the United Kingdom, 1861–19571,” *Economica*, vol. 25, no. 100, 1958, doi: 10.1111/j.1468-0335.1958.tb00003.x

[24] Carranza L., Galdon-Sanchez J. E., and Gomez-Biscarri J., “Understanding the Relationship between Financial Development and Monetary Policy,” *Rev Int Econ*, vol. 18, no. 5, 2010, doi: 10.1111/j.1467-9396.2010.00926.x

[25] Maiga F. K., “Impact of interest rate on economic growth in Nigeria,” *Pyrex journal of business and finance management research*, vol. 3, no. 3, pp. 98–111, 2017.

[26] Özen E., Özdemir L., and Grima S., “The Relationship between the Exchange Rate, Interest Rate and Inflation: The Case of Turkey,” *Scientific Annals of Economics and Business*, vol. 67, no. 2, 2020, doi: 10.47743/SAEB-2020-0014

[27] Bernanke B. S., Gertler M., and Gilchrist S., “Chapter 21 The financial accelerator in a quantitative business cycle framework,” *Handbook of Macroeconomics*, vol. 1, no. PART C. 1999. doi: 10.1016/S1574-0048(99)10034-X

[28] Ali A. and Bibi C., “Determinants of Social Progress and its Scenarios under the role of Macroeconomic Instability: Empirics from Pakistan,” *Munich Personal Repec Archive*, vol. 55, no. 72920, 2016.

[29] Audi M., Ali A., and Al-Masri R., “Determinants of Advancement in Information Communication Technologies and its Prospect under the role of Aggregate and Disaggregate Globalization,” *Scientific Annals of Economics and Business*, vol. 69, no. 2, pp. 191–215, Jun. 2022, doi: 10.47743/saeb-2022-0009

[30] Bruno M. and Easterly W., “Inflation crises and long-run growth,” *J Monet Econ*, vol. 41, no. 1, 1998, doi: 10.1016/S0304-3932(97)00063-9

[31] Easterly W. and Fischer S., “Inflation and the Poor,” *J Money Credit Bank*, vol. 33, no. 2, 2001, doi: 10.2307/2673879

[32] Yaya K., “Testing the Long-Run Fisher Effect in Selected African Countries: Evidence from ARDL Bounds Test,” *Int J Econ Finance*, vol. 7, no. 12, 2015, doi: 10.5539/ijef.v7n12p168

[33] Hayat M. A. et al., “Investigating the Causal Linkages among Inflation, Interest Rate, and Economic Growth in Pakistan under the Influence of COVID-19 Pandemic: A Wavelet Transformation Approach,” *Journal of Risk and Financial Management*, vol. 14, no. 6, 2021, doi: 10.3390/jrfm14060277

[34] CEYLAN F., CEYLAN Işıl EREM, TÜZÜN Osman, and EKİNCİ Ramazan, “The Casual Relationship Between Inflation Uncertainty and Interest Rate in Turkey: Rolling Window Causality Test,” *Mehmet Akif Ersoy Üniversitesi İktisadi ve İdari Bilimler Fakültesi Dergisi*, vol. 5, no. 3, 2018.

[35] Asgharpur H., Kohnehshahri L. A., Karami Bangor A., and School B., “THE RELATIONSHIPS BETWEEN INTEREST RATES AND INFLATION CHANGES: AN ANALYSIS OF LONG-TERM INTEREST RATE DYNAMICS IN DEVELOPING COUNTRIES,” 2007.

[36] Mirza A. and Rashidi M., “Causal Relationship between Interest Rate and Inflation Rate: A study of SAARC Economies,” *Kardan Journal of Economics and Manangement Sciences*, 2018, doi: 10.31841/kjems.2021.80

[37] Nezhad M. Z. and Zarea R., “Investigating the Causality Granger Relationship between the Rates of Interest and Inflation in Iran,” *Journal of Social Sciences*, vol. 3, no. 4, 2007, doi: 10.3844/jssp.2007.237.244

[38] Amaefula C. G., “Long-Run Relationship between Interest Rate and Inflation: Evidence from Nigeria,” *IOSR Journal of Economics and Finance*, vol. 7, 2016.

[39] MMASI B., “An Investigation of the Relationship Between Interest Rate and Inflation in Kenya,” University of Nairobi, 2013.

[40] Saymeh A. A. F. and Orabi M. M. A., “The Effect of Interest Rate, Inflation Rate, GDP, on Real Economic Growth Rate in Jordan,” *Asian Economic and Financial Review*, vol. 3, no. 3, 2013.

[41] Maziars M., “A review of the Granger-causality fallacy,” *The Journal of Philosophical Economics Reflections on Economic and Social Issues*, vol. 8, no. 2, 2015.

[42] Heckman J. J. and Pinto R., “Causality and Econometrics,” *SSRN Electronic Journal*, 2022, doi: 10.2139/ssrn.4048252

[43] Mansournia M. A., “Sample size considerations are needed for the causal analyses of existing databases,” *Journal of Clinical Epidemiology*, vol. 141. 2022. doi: 10.1016/j.jclinepi.2021.09.024 34551318

[44] Heidel R. E., “Causality in Statistical Power: Isomorphic Properties of Measurement, Research Design, Effect Size, and Sample Size,” *Scientifica*, vol. 2016. 2016. doi: 10.1155/2016/8920418 27073717 PMC4814708

[45] Wu L., Adebayo T. S., Yue X. G., and Umut A., “The role of renewable energy consumption and financial development in environmental sustainability: implications for the Nordic Countries,” *International Journal of Sustainable Development and World Ecology*, vol. 30, no. 1, 2023, doi: 10.1080/13504509.2022.2115577

[46] Benhmad F., “Dynamic cyclical comovements between oil prices and US GDP: A wavelet perspective,” *Energy Policy*, vol. 57, 2013, doi: 10.1016/j.enpol.2013.01.017

[47] Fan Q., Yang H., Li G., and Zhao J., “Suppressing carrier removal error in the Fourier transform method for interferogram analysis,” *Journal of Optics*, vol. 12, no. 11, 2010, doi: 10.1088/2040-8978/12/11/115401

[48] Hanif M., Zaheer S., and Iqbal J., “Time-Frequency Analysis of Determinants of Inflation Rate in Pakistan,” 11, 22AD.

[49] Yogo M., “Measuring business cycles: A wavelet analysis of economic time series,” *Econ Lett*, vol. 100, no. 2, 2008, doi: 10.1016/j.econlet.2008.01.008

[50] Crowley P. M. M., “Long Cycles in Growth: Explorations Using New Frequency Domain Techniques with US Data,” *SSRN Electronic Journal*, 2012, doi: 10.2139/ssrn.1573641

[51] Tiwari A. K., Olayeni R. O., Olofin S. A., and Chang T., “The Indian inflation–growth relationship revisited: robust evidence from time–frequency analysis,” *Appl Econ*, vol. 51, no. 51, 2019, doi: 10.1080/00036846.2019.1616065

[52] Easterly W., “What did structural adjustment adjust? The association of policies and growth with repeated IMF and World Bank adjustment loans,” *J Dev Econ*, vol. 76, no. 1, 2005, doi: 10.1016/j.jdeveco.2003.11.005

